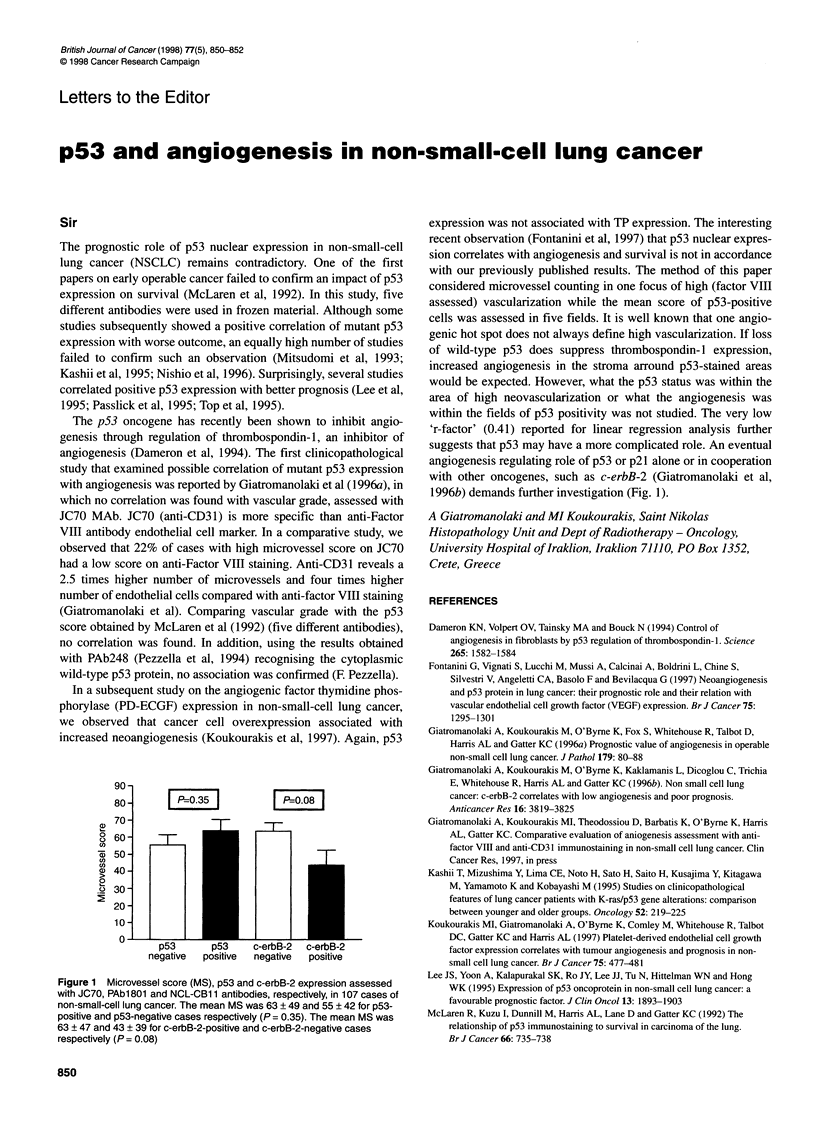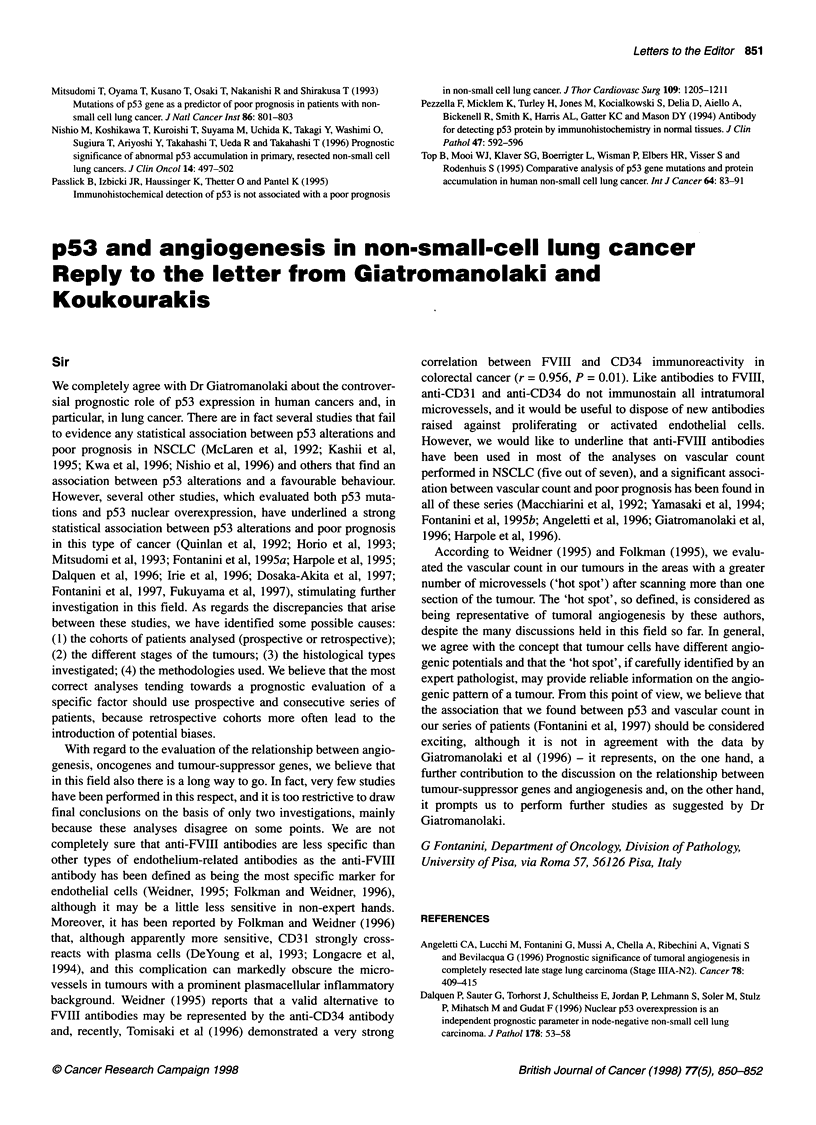# p53 and angiogenesis in non-small-cell lung cancer.

**DOI:** 10.1038/bjc.1998.138

**Published:** 1998-03

**Authors:** A. Giatromanolaki, M. I. Koukourakis


British Journal of Cancer (1998) 77(5), 850–852
© 1998 Cancer Research Campaign

Letters to the Editor

### p53 and angiogenesis in non-small-cell lung cancer

### Sir

The prognostic role of p53 nuclear expression in non-small-cell lung cancer (NSCLC) remains contradictory. One of the first papers on early operable cancer failed to confirm an impact of p53 expression on survival (McLaren et al, 1992). In this study, five different antibodies were used in frozen material. Although some studies subsequently showed a positive correlation of mutant p53 expression with worse outcome, an equally high number of studies failed to confirm such an observation (Mitsudomi et al, 1993; Kashii et al, 1995; Nishio et al, 1996). Surprisingly, several studies correlated positive p53 expression with better prognosis (Lee et al, 1995; Passlick et al, 1995; Top et al, 1995).

The *p53* oncogene has recently been shown to inhibit angiogenesis through regulation of thrombospondin-1, an inhibitor of angiogenesis (Dameron et al, 1994). The first clinicopathological study that examined possible correlation of mutant p53 expression with angiogenesis was reported by Giatromanolaki et al (1996*a*), in which no correlation was found with vascular grade, assessed with JC70 MAb. JC70 (anti-CD31) is more specific than anti-Factor VIII antibody endothelial cell marker. In a comparative study, we observed that 22% of cases with high microvessel score on JC70 had a low score on anti-Factor VIII staining. Anti-CD31 reveals a 2.5 times higher number of microvessels and four times higher number of endothelial cells compared with anti-factor VIII staining (Giatromanolaki et al). Comparing vascular grade with the p53 score obtained by McLaren et al (1992) (five different antibodies), no correlation was found. In addition, using the results obtained with PAb248 (Pezzella et al, 1994) recognising the cytoplasmic wild-type p53 protein, no association was confirmed (F. Pezzella).

In a subsequent study on the angiogenic factor thymidine phosphorylase (PD-ECGF) expression in non-small-cell lung cancer, we observed that cancer cell overexpression associated with increased neoangiogenesis (Koukourakis et al, 1997). Again, p53 expression was not associated with TP expression. The interesting recent observation (Fontanini et al, 1997) that p53 nuclear expression correlates with angiogenesis and survival is not in accordance with our previously published results. The method of this paper considered microvessel counting in one focus of high (factor VIII assessed) vascularization while the mean score of p53-positive cells was assessed in five fields. It is well known that one angiogenic hot spot does not always define high vascularization. If loss of wild-type p53 does suppress thrombospondin-1 expression, increased angiogenesis in the stroma arround p53-stained areas would be expected. However, what the p53 status was within the area of high neovascularization or what the angiogenesis was within the fields of p53 positivity was not studied. The very low 'r-factor' (0.41) reported for linear regression analysis further suggests that p53 may have a more complicated role. An eventual angiogenesis regulating role of p53 or p21 alone or in cooperation with other oncogenes, such as *c-erbB*-2 (Giatromanolaki et al, 1996*b*) demands further investigation (Fig. 1).

**Figure 1** Microvessel score (MS), p53 and c-erbB-2 expression assessed with JC70, PAb1801 and NCL-CB11 antibodies, respectively, in 107 cases of non-small-cell lung cancer. The mean MS was 63 ± 49 and 55 ± 42 for p53-positive and p53-negative cases respectively ($P = 0.35$). The mean MS was 63 ± 47 and 43 ± 39 for c-erbB-2-positive and c-erbB-2-negative cases respectively ($P = 0.08$)

*A Giatromanolaki and MI Koukourakis, Saint Nikolas Histopathology Unit and Dept of Radiotherapy – Oncology, University Hospital of Iraklion, Iraklion 71110, PO Box 1352, Crete, Greece*

### REFERENCES

Dameron KN, Volpert OV, Tainsky MA and Bouck N (1994) Control of angiogenesis in fibroblasts by p53 regulation of thrombospondin-1. *Science* **265**: 1582–1584

Fontanini G, Vignati S, Lucchi M, Mussi A, Calcinai A, Boldrini L, Chine S, Silvestri V, Angeletti CA, Basolo F and Bevilacqua G (1997) Neoangiogenesis and p53 protein in lung cancer: their prognostic role and their relation with vascular endothelial cell growth factor (VEGF) expression. *Br J Cancer* **75**: 1295–1301

Giatromanolaki A, Koukourakis M, O'Byrne K, Fox S, Whitehouse R, Talbot D, Harris AL and Gatter KC (1996*a*) Prognostic value of angiogenesis in operable non-small cell lung cancer. *J Pathol* **179**: 80–88

Giatromanolaki A, Koukourakis M, O'Byrne K, Kaklamanis L, Dicoglou C, Trichia E, Whitehouse R, Harris AL and Gatter KC (1996*b*). Non small cell lung cancer: c-erbB-2 correlates with low angiogenesis and poor prognosis. *Anticancer Res* **16**: 3819–3825

Giatromanolaki A, Koukourakis MI, Theodossiou D, Barbatis K, O'Byrne K, Harris AL, Gatter KC. Comparative evaluation of aniogenesis assessment with anti-factor VIII and anti-CD31 immunostaining in non-small cell lung cancer. Clin Cancer Res, 1997, in press

Kashii T, Mizushima Y, Lima CE, Noto H, Sato H, Saito H, Kusajima Y, Kitagawa M, Yamamoto K and Kobayashi M (1995) Studies on clinicopathological features of lung cancer patients with K-ras/p53 gene alterations: comparison between younger and older groups. *Oncology* **52**: 219–225

Koukourakis MI, Giatromanolaki A, O'Byrne K, Comley M, Whitehouse R, Talbot DC, Gatter KC and Harris AL (1997) Platelet-derived endothelial cell growth factor expression correlates with tumour angiogenesis and prognosis in non-small cell lung cancer. *Br J Cancer* **75**: 477–481

Lee JS, Yoon A, Kalapurakal SK, Ro JY, Lee JJ, Tu N, Hittelman WN and Hong WK (1995) Expression of p53 oncoprotein in non-small cell lung cancer: a favourable prognostic factor. *J Clin Oncol* **13**: 1893–1903

McLaren R, Kuzu I, Dunnill M, Harris AL, Lane D and Gatter KC (1992) The relationship of p53 immunostaining to survival in carcinoma of the lung. *Br J Cancer* **66**: 735–738

Mitsudomi T, Oyama T, Kusano T, Osaki T, Nakanishi R and Shirakusa T (1993) Mutations of p53 gene as a predictor of poor prognosis in patients with non-small cell lung cancer. *J Natl Cancer Inst* **86**: 801–803

Nishio M, Koshikawa T, Kuroishi T, Suyama M, Uchida K, Takagi Y, Washimi O, Sugiura T, Ariyoshi Y, Takahashi T, Ueda R and Takahashi T (1996) Prognostic significance of abnormal p53 accumulation in primary, resected non-small cell lung cancers. *J Clin Oncol* **14**: 497–502

Passlick B, Izbicki JR, Haussinger K, Thetter O and Pantel K (1995) Immunohistochemical detection of p53 is not associated with a poor prognosis in non-small cell lung cancer. *J Thor Cardiovasc Surg* **109**: 1205–1211

Pezzella F, Micklem K, Turley H, Jones M, Kocialkowski S, Delia D, Aiello A, Bickenell R, Smith K, Harris AL, Gatter KC and Mason DY (1994) Antibody for detecting p53 protein by immunohistochemistry in normal tissues. *J Clin Pathol* **47**: 592–596

Top B, Mooi WJ, Klaver SG, Boerrigter L, Wisman P, Elbers HR, Visser S and Rodenhuis S (1995) Comparative analysis of p53 gene mutations and protein accumulation in human non-small cell lung cancer. *Int J Cancer* **64**: 83–91